# Long term follow-up and further molecular and histopathological studies in the LGMD1F sporadic *TNPO3*-mutated patient

**DOI:** 10.1186/s40478-018-0648-4

**Published:** 2018-12-19

**Authors:** Sara Gibertini, Alessandra Ruggieri, Simona Saredi, Franco Salerno, Flavia Blasevich, Laura Napoli, Maurizio Moggio, Vincenzo Nigro, Lucia Morandi, Lorenzo Maggi, Marina Mora

**Affiliations:** 1 Neuromuscular Diseases and Neuroimmunology Unit, Fondazione IRCCS Istituto Neurologico Carlo Besta, Via Temolo 4, 20126 Milan, Italy; 2 TIGEM (Telethon Institute of Genetics and Medicine), Naples, Italy; 3 Dipartimento di Biochimica Biofisica e Patologia Generale, Università degli Studi della Campania “Luigi Vanvitelli”, Naples, Italy; 4 Neuromuscular and Rare Diseases Unit, Department of Neurosciences, Fondazione IRCCS Ca’ Granda, Ospedale Maggiore Policlinico, Milan, Italy

**Keywords:** TNPO3, Transportin 3, LGMD1F

## Introduction

Limb girdle muscular dystrophies (LGMD) are a large group of muscular disorders, with progressive shoulder and pelvic muscle weakness as the most relevant feature. They are classified as autosomal dominant (LGMD1) and autosomal recessive (LGMD2) forms. Up to now, eight genetically defined LGMD1 subtypes (LGMD1 A-H) have been identified [1, 9].

In 2001, the clinical and morphological phenotype of a novel form of LGMD type 1, affecting 32 subjects in a large Spanish family, was described [5]. According to subsequent molecular studies, the disease was demonstrated to be linked to the novel chromosomal locus 7q32.1–32.2. This genetically distinct form of autosomal dominant-LGMD was classified as LGMD1F [10] (OMIM #608423). Recently, using a whole genome sequencing approach, the causative mutation of the LGMD1F was identified in the termination codon of *TNPO3,* the gene coding for transportin 3. Molecular results at DNA, RNA and protein levels as well as morphological findings supported the pathogenic role of this mutation in LGMD1F [8]. Investigation by next-generation sequencing in further 4 members of the Spanish family, originating from Italy, confirmed the mutation in *TNPO3* [13]. Up to now, beside this Italo-Spanish family, only one sporadic LGMD patient has been identified with a heterozygous point mutation in the *TNPO3* gene [13].

In this patient we now report the long term clinical and radiological follow-up, morphological and immunochemical studies on patient muscle biopsy, and molecular studies by Real Time PCR and by cell transfection with the mutant cDNA.

## Case report

The patient was investigated at our institute when he was 38 years old because of slowly progressive difficulty in walking and climbing stairs presenting since the age of 35 years. No familial occurrence of neuromuscular disorders or consanguinity was referred.

On neurological examination scapular, anterior and posterior thigh muscle atrophy were observed. Assessment of muscle strength using the British Medical Research Council (MRC) scale, showed weakness of shoulder girdle muscles (with arm flexion and abduction possible against gravity until 90°), without scapular winging, inferior trapezius (2/5), arm extensors (2/5), elbow flexors (3/5), hip flexors (3/5), hip extensors (2/5), knee flexors and extensors (4/5), dorsal foot extensors (4/5). No cranial nerve involvement was observed. Joint contractures and skeletal deformities were not detected. The patient presented a waddling gait with an increased lumbar lordosis and was unable to get up from the floor. Functional ability of upper limbs was 3 according to Brooke scale (from 1: normal; to 6: no function for upper extremity) [2] and lower limb function was 3 according to Vignos scale (1: able to climb stairs without help; to 10: bedridden for lower limb function) [14]. CK was only slightly increased (253 UI/l). EMG showed myopathic findings in all tested muscles with fibrillation potentials and positive sharp waves. On muscle CT scan, moderate fatty changes were found in bilateral quadriceps and hamstrings and medial gastrocnemius. Respiratory and cardiac functions were normal. Symptoms progressively worsened in the following years, loosing the ability to climb stairs at the age of 45.

A muscle biopsy from the left quadriceps, taken at age 38, displayed fibre size variability, a few central nuclei, scattered degenerative fibres (Fig. 2), few cytochrome oxidase-negative fibres, and ragged red appearing fibres that, although rare (about 1%) were above the expected number in a 38 years old man. Immunostaining for dystrophin, sarcoglycans, caveolin 3, and alpha-dystroglycan, was normal, as well as dysferlin and calpain 3 immunoblotting. Respiratory chain activity and mitochondrial DNA analysis by Southern blot were normal.

By next generation sequencing analysis, a heterozygous G > A transition (c.G2453A) in exon 20 of the *TNPO3* gene was found (reported in exon 21 in the original paper) [13]. The G > A point mutation changes the arginine in position 818 with a glutamine in a highly conserved residue, predicted to be damaging by all the used bioinformatic tools. This mutation is now listed in dbSNP (rs587777431) and it is present in gnomAD (The Genome Aggregation Database) with a population frequency of 0.00004215. This variant was not found in the two healthy sisters.

After publication of the original report [13], we extensively reassessed muscle biopsy, clinical features and radiologic findings in the patient and performed transfection studies to characterize the mutation.

**Fig. 1:**
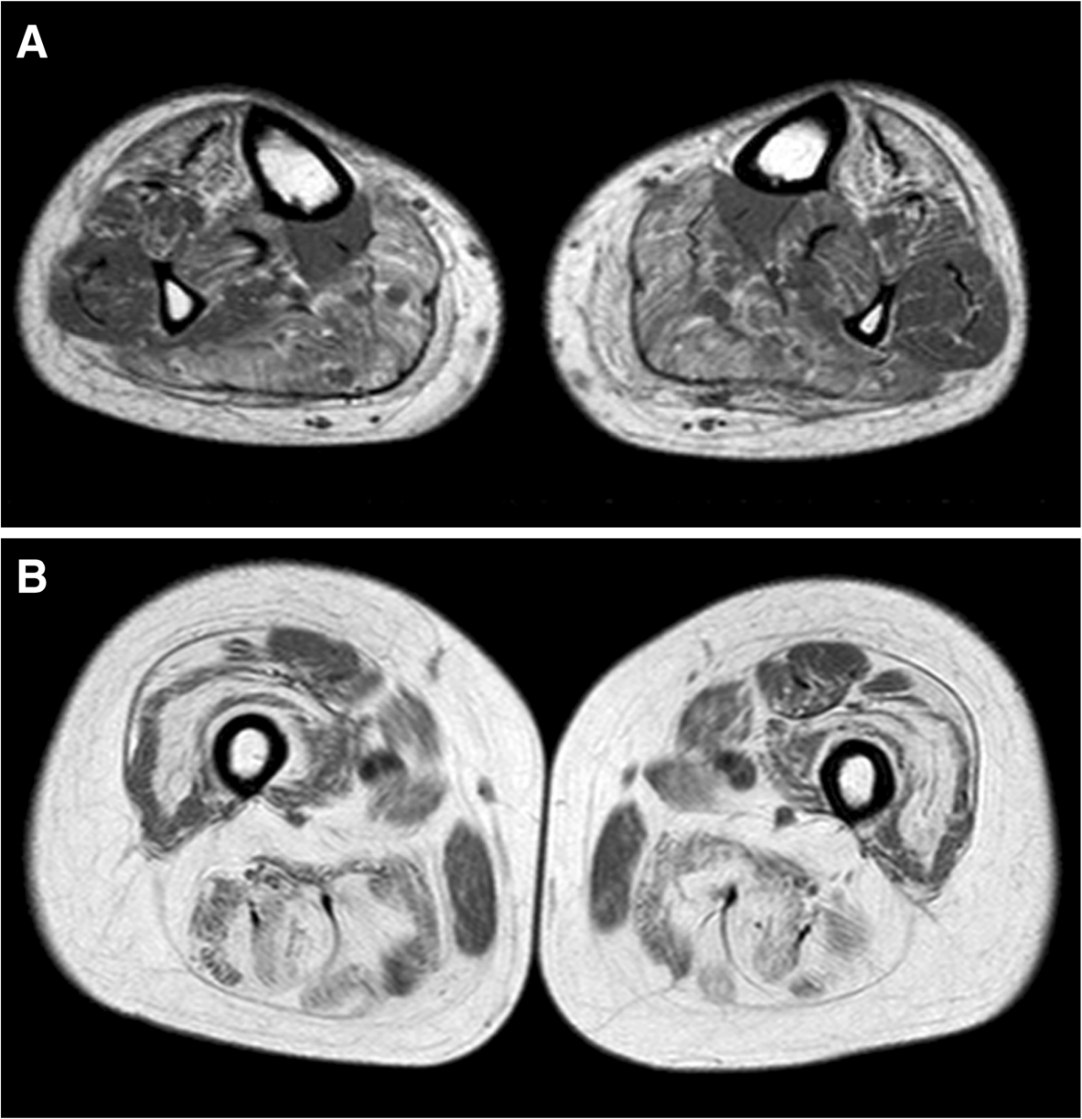
T1-weighted muscle MRI at leg (**a**) and thigh (**b**) level. In the leg symmetrical fatty changes are more evident in medial and lateral gastrocnemius and, to a lesser degree, in tibialis anterior muscles (**a**). In the thigh a diffuse fatty substitution is present, with relative sparing of gracilis and rectus femoris muscles (**b**)

On his last visit, at age 54, the patient showed a severe waddling gait and was able to walk only with assistance of the caregiver. The patient needed a wheelchair for longer distances. He also required assistance for dressing, bathing and getting up from the chair. Neurological examination showed mild cranial nerve involvement, including tongue weakness, eyelid ptosis and minimal ophthalmoparesis in the lateral gaze. Bilateral elbow joint laxity and left Achilles’ tendon retraction were observed. Beevor’s sign (upward movement of the umbilicus on flexing the neck in supine position) was present. Assessment of muscle strength showed weakness of neck extensors (3/5) and flexors (4/5), arm flexion and abduction, both possible until 20–30° and without scapular winging, inferior trapezius (1/5), elbow flexors and extensors (2/5), finger flexors and extensors (4/5), hip flexors, adductors, extensors and abductors (1/5), knee extensors (right: 1/5; left: 2/5), dorsal foot extensors, especially tibialis anterior (left: 3/5; right: 4/5). Functional ability of upper and lower limbs according to Brooke and Vignos scales [2, 14] was 4 and 6, respectively. Lower limb muscle MRI at 54 years revealed an almost complete and symmetrical fatty substitution of thigh muscles, with relative sparing of gracilis and rectus femoris (Fig. 1); medial and lateral gastrocnemius and, to a lesser extent, tibialis anterior and soleus, were the most involved muscles in the legs (Fig. 1). Pulmonary function tests showed a moderate decline of forced vital capacity (60% of predicted value); nocturnal saturimetry was normal. No cardiac involvement was detected.

**Fig. 2:**
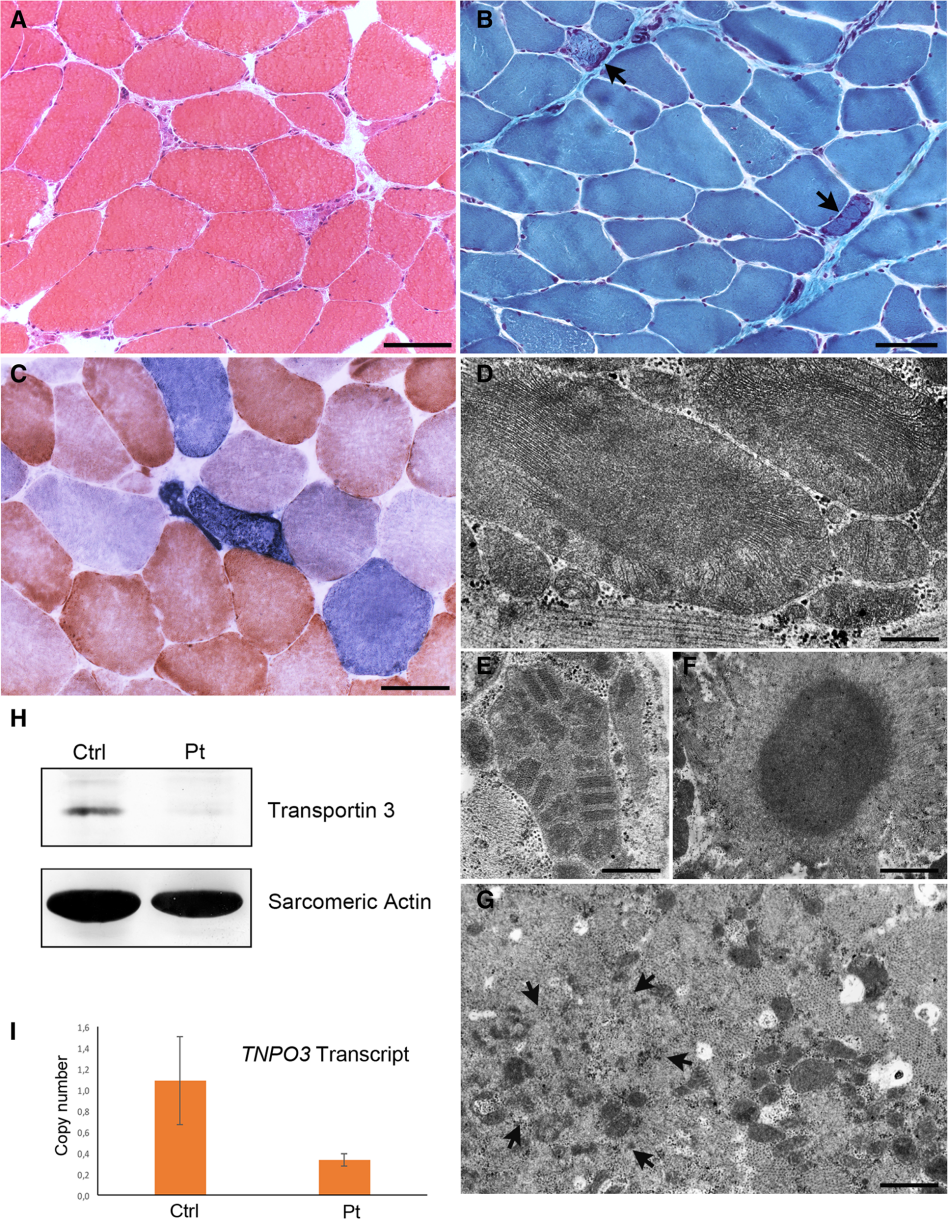
(**a**) **h** & **e**, (**b**) Gomori Trichrome and (**c**) COX/SDH staining showing, in the first biopsy (**a**), a few atrophic degenerating fibres, and, in the second biopsy (**b**, **c**), ragged red-like fibres (arrows), some of which appear intensely positive to SDH (COX-positive fibres stain brown, while COX-deficient fibres stain blue due to preserved SDH activity). Bars = 50 μm. (**d**-**g**) Electron microscopy images of first (**d**) and second biopsy (**e**-**g**) showing mitochondria of abnormally large size containing densely packed cristae (D) or paracrystalline inclusions (**e**), a cytoplasmic body (**f**), and myofibrillar disorganization (arrows) (**g**). Bar G = 250 nm; E = 500 nm; F,G = 1 μm. **h** Western blot showing reduced intensity of the transportin 3 band; (**i**) Real time PCR showing decreased *TNPO3* transcript levels

Reassessment of morphology on a second muscle biopsy of the right quadriceps, taken at the Ospedale Maggiore Policlinico of Milano at age 40, showed no overall progression of histopathological features compared to the biopsy at age 38 years. These included the presence of ragged red and COX-negative/SDH-positive fibres (Fig. 2) (about 1% as in the first biopsy), and of degenerating fibres. Electron microscopy confirmed the presence of non-specific degenerative aspects, and showed small areas of myofibrillar disorganization in a few fibres, cytoplasmic bodies in the subsarcolemmal region of two fibres, mitochondrial abnormalities in several fibres (Fig. 2), but no clear rimmed vacuoles or nuclear alterations. Mitochondrial abnormalities consisted of increased number and size of these organelles that appeared often as gigantic and contained densely packed cristae, paracrystalline inclusions or dark homogeneous inclusions (Fig. 2).

Western blot analysis of muscle homogenate using antibodies against transportin 3 showed that the band corresponding to the protein was of greatly reduced intensity in the patient compared to a control subject (Fig. 2).

A Real Time PCR assay, carried out to quantify transcript levels of *TNPO3* in the patient, revealed a reduction of more than 50% in the patient mRNA compared to controls (Fig. 2).

**Fig. 3:**
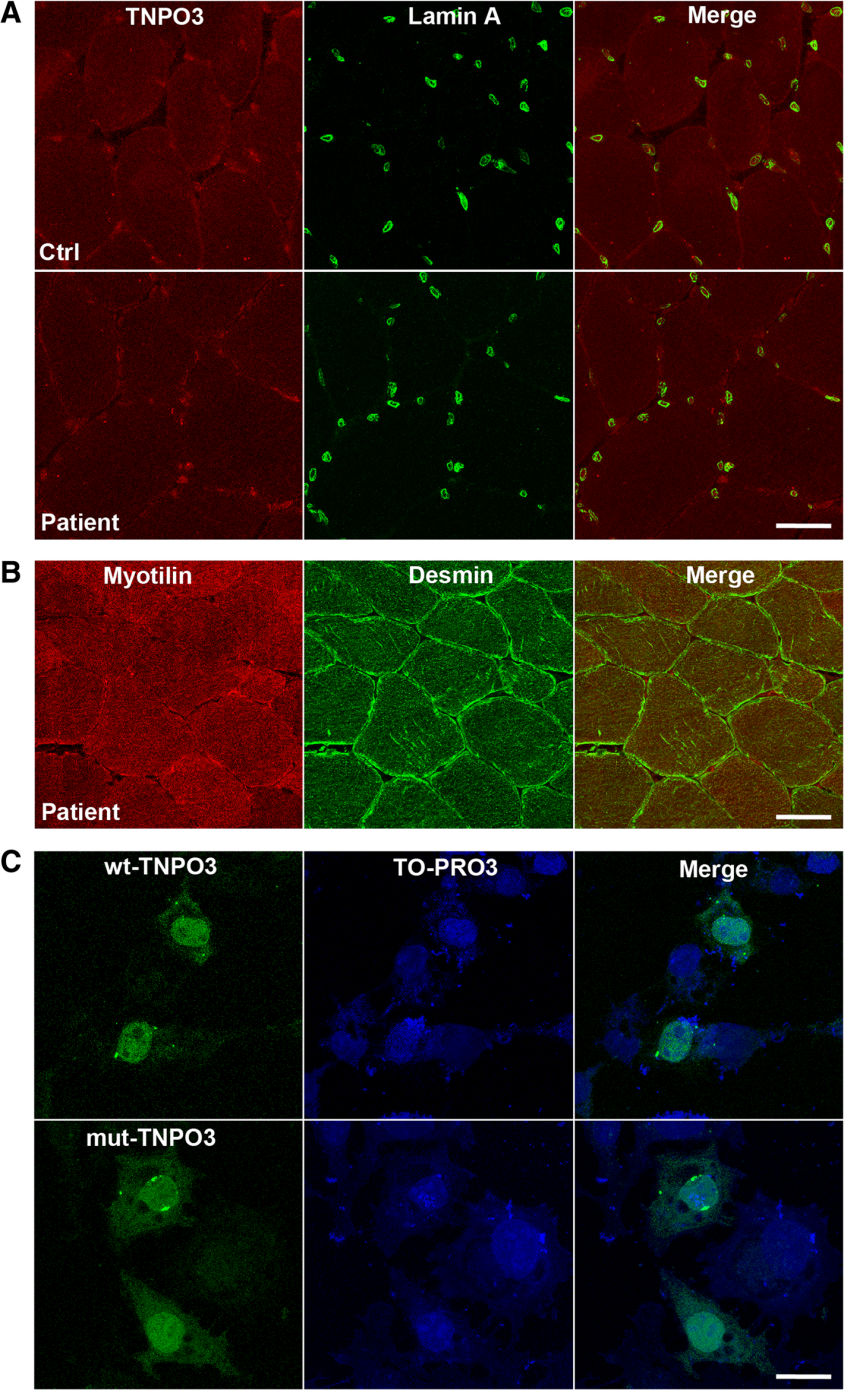
**a** Co-localization of transportin 3 and lamin A, and (**b**) of desmin and myotilin in the second muscle biopsy showing that, similarly to control muscle, transportin 3 is localized in the patient muscle mainly at the nuclei; and that desmin and myotilin are normally expressed. Bars = 50 μm. **c** COS7 cells transfected with wild-type or mutant *TNPO3* cDNA showing correct localization to the nucleus of both mutant and wild-type transportin 3. Bar = 10 μm

By immunohistochemistry, transportin 3 localized normally at the muscle fibre nuclei, and myofibrillar desmin- or myotilin-positive aggregates typical of myofibrillar myopathies, were not observed (Fig. 3). Immunohistochemical evaluation of autophagy using antibodies against EEA1 (early endosome antigen), LC3 (microtubule-associated protein 1 light chain 3), LAMP2 (marker of lysosomes and late endosomes), P62 (Sequestosome-1), FK2 (ubiquitinylated proteins), and BAG3 (BCL associated athanogene-3) failed to show autophagy activation (not shown). Transfection of COS7 cells with a mutant *TNPO3* cDNA containing the exon 20 G > A transition, or with the wild-type *TNPO3* cDNA showed correct localization to the nucleus of both mutant and wild-type transportin 3 (Fig. 3). Immunostaining of transfected cells with anti-p62 or anti-LC3 failed to show any difference between wild-type and mutant *TNPO3*-transfected cells.

## Discussion

LGMD1F, reported so far only in the large Italo-Spanish kindred, is clinically characterized by pelvic and shoulder girdle weakness, with a wide variability in the age at onset, spanning from 1 to 58 years. Individuals with juvenile onset presented severe and rapid progression of the disease involving proximal and distal limb muscles and leading to early loss of autonomous walking. Patients with adult onset disease manifested a slow progression of symptoms and persistent ability to walk. Other aspects of the clinical phenotype considered as specific indicators of LGMD1F are dysphagia, arachnodactyly with or without finger contractures, and dysarthria [11]. Our sporadic case is similar to the patients of the Italo-Spanish family with adult onset of symptoms and moderate progression of weakness. Differently from the family patients, he does not manifest any of the adjunctive symptoms described and suggested as specific of LGMD1F. Some differences were also observed at muscle MRI. Compared to most affected patients reported by Melià and colleagues [8], our patient showed a more diffuse involvement of thigh muscles, with relative and selective sparing of gracilis and rectus femoris, and less severe involvement of lower leg muscles.

Like in the Italo-Spanish family, myofibrillar abnormalities, although minor, as well as mitochondrial abnormalities [3, 4], were observed in muscle biopsies of our patient. No progression of histopathological features was observed in the patient muscle, however the interval between the two biopsies was only two years; furthermore being both biopsies from long time ago, the histological features cannot be correlated to the clinical features observed in the most recent clinical follow-up.

A possible mitochondrial dysfunction has been hypothesized in myofibrillar myopathies such as the desmin- or the filamin-mutated myopathies and mitochondrial abnormalities have been interpreted as a secondary phenomenon and an early histological sign [6, 12]. A role for transportin 3 in mitochondrial function cannot be ruled out and will need further studies and in patients with different *TNPO3* mutations to shed light on its function in muscle disease. Transportin 3, being implicated in the translocation of splice regulators to the nucleoplasm and in pre-mRNA processing [7], could indeed play a role in the maturation of RNAs coding for mitochondrial or myofibrillar proteins.

The missense change in our patient, unlike the reported c.2771del, does not affect protein localization, but causes a reduction in *TNPO3* transcript, likely due to messenger instability, and a consequent reduction of the protein. The affected residue R818 is in a helix region towards the C-terminus of the protein, the role of which is not precisely defined. The overall efficiency of transportin 3 is likely insufficient to mediate nuclear translocation of its ligands in our patient.

On the whole our study provides further histopathological, molecular and clinical insights on this still imprecise muscular dystrophy.
